# Perceptive SARS-CoV-2 End-To-End Ultrasound Video Classification through X3D and Key-Frames Selection

**DOI:** 10.3390/bioengineering10030282

**Published:** 2023-02-21

**Authors:** Marco Gazzoni, Marco La Salvia, Emanuele Torti, Gianmarco Secco, Stefano Perlini, Francesco Leporati

**Affiliations:** 1 Department of Electrical, Computer and Biomedical Engineering, University of Pavia, 27100 Pavia, Italy; 2 Emergency Medicine Unit and Emergency Medicine Postgraduate Training Program, Department of Internal Medicine, University of Pavia, IRCCS Policlinico San Matteo Foundation, 27100 Pavia, Italy

**Keywords:** video classification, SARS-CoV-2, Lung Ultrasound, deep learning

## Abstract

The SARS-CoV-2 pandemic challenged health systems worldwide, thus advocating for practical, quick and highly trustworthy diagnostic instruments to help medical personnel. It features a long incubation period and a high contagion rate, causing bilateral multi-focal interstitial pneumonia, generally growing into acute respiratory distress syndrome (ARDS), causing hundreds of thousands of casualties worldwide. Guidelines for first-line diagnosis of pneumonia suggest Chest X-rays (CXR) for patients exhibiting symptoms. Potential alternatives include Computed Tomography (CT) scans and Lung UltraSound (LUS). Deep learning (DL) has been helpful in diagnosis using CT scans, LUS, and CXR, whereby the former commonly yields more precise results. CXR and CT scans present several drawbacks, including high costs. Radiation-free LUS imaging requires high expertise, and physicians thus underutilise it. LUS demonstrated a strong correlation with CT scans and reliability in pneumonia detection, even in the early stages. Here, we present an LUS video-classification approach based on contemporary DL strategies in close collaboration with Fondazione IRCCS Policlinico San Matteo’s Emergency Department (ED) of Pavia. This research addressed SARS-CoV-2 patterns detection, ranked according to three severity scales by operating a trustworthy dataset comprising ultrasounds from linear and convex probes in 5400 clips from 450 hospitalised subjects. The main contributions of this study are related to the adoption of a standardised severity ranking scale to evaluate pneumonia. This evaluation relies on video summarisation through key-frame selection algorithms. Then, we designed and developed a video-classification architecture which emerged as the most promising. In contrast, the literature primarily concentrates on frame-pattern recognition. By using advanced techniques such as transfer learning and data augmentation, we were able to achieve an F1-Score of over 89% across all classes.

## 1. Introduction

The SARS-CoV-2 virus, which originated in China in 2019, has spread globally and is highly contagious [1]. It has a variable incubation period, during which infected individuals may exhibit a range of symptoms including fever, dry cough, fatigue, and difficulty breathing [2]. However, some infected people may not show any symptoms at all. The virus can also cause a variety of clinical presentations, including bilateral multi-focal interstitial pneumonia, which can progress to acute respiratory distress syndrome (ARDS).

Lung UltraSound testing aids in visualising and quantifying pulmonary involvement, typically retaining the white lung pattern or bilateral submantellar-subpleural consolidations [3,4]. The primary method of SARS-CoV-2 diagnosis is the nasopharyngeal swab and the combined IgM-IgG antibody test [5]. The nasopharyngeal swab relies on real-time reverse transcription-polymerase chain reactions (rRT-PCR). Therefore, the main drawbacks include long response times and shortages in reagents and other specific laboratory supplies. On the other hand, the IgM-IgG antibody test features a lower sensitivity than rRT-PCR, yielding false-negative results in the early phases of the infection. Specifically, the disease begins with mild symptoms but can rapidly progress to severe forms leading to fatal consequences from multi-organ failure. Hence, the fast progression highlights the importance of developing a human-sensible perceptive device that can detect the disease’s presence and assess its degree of severity.

In the literature, the first line diagnosis of pneumonia exploits X-rays (CXR) [6], which also enables fast first-aid for patients showing pneumonia symptoms. The literature also indicates that Computer Tomography (CT) [7] scans and Lung UltraSound (LUS) [8] represent an alternative to CXR. 

Different studies compared these techniques highlighting that CT and LUS outperform CXR [9,10,11]. The main conclusions from studies concerning these methodologies state that: first, both LUS and CT scans are significantly better first-line diagnostic tools than CXR, whose main drawback is poor sensitivity; second, although ultrasonography is a cost-effective, radiation-free, and promising tool, it must be performed by a highly skilled radiographer to achieve accurate results. Furthermore, LUS effectively performed at a bedside in approximately 13 min yielded a higher sensitivity than that of CXR. This makes it comparable to other CT imaging tools with its cost being significantly lower than those of the other two solutions.

In this context, academia evaluated different Deep Learning (DL) models to automatically expose the presence of SARS-CoV-2 from medical images [12]. Several works considered SARS-CoV-2 diagnosis exploiting LUS [8,10,11,12]. All these studies assessed a single frame extracted from the video assembled by the LUS probe. It is essential to highlight that an expert manually selected the frame to be classified to ensure that the main patterns were present in the image. This aspect limits the applicability of these procedures since the final results strictly depend on the frames extracted, and few works address this issue. In particular, researchers [13] evaluated a Two-Stream Inflated 3D ConvNet (I3D) to perform the end-to-end video classification. The results comprise precision, recall and F1-Score on the A and B lines LUS patterns. Consequently, this network cannot diagnose SARS-CoV-2 directly.

On the other hand, another investigation [14] conceived a network based on Convolutional Neural Network (CNN) and Long-Short Term Memory (LSTM) cells. This method features accuracy, precision, and recall at approximately 92% in the most promising configuration. Regardless, the study does not rank SARS-CoV-2 pneumonia severity but only differentiates viral and bacterial cases of pneumonia from a healthy lung. Besides, it operates a sequence of features extracted from the frames with a CNN. Hence, it does not address the end-to-end video classification of an LUS clip. Eventually, the literature retains a final study [15] that compared a Multi-Layer Perceptron (MLP) network, the EfficentNet and the Vision Transformer (ViT). The study found that the EfficientNet outperforms the other techniques measuring 96% accuracy. Nonetheless, even if the paper addresses video classification, the networks target the classification of a single frame without explaining how the authors chose the clip’s frame.

Here, we propose an investigation comparing different video classification methodologies, resulting in the X3D network as the most performing one. Accordingly, this manuscript’s main contributions are:

- Analysis of different key-frame selection strategies to perform LUS clip summarisation and extract meaningful content, resulting in fewer data to be elaborated and faster diagnosis. We evaluated the key-frame selection approaches, training benchmark architectures on the selected frames and testing on frames that experienced physicians extracted, containing pneumonia scoring patterns that the end-to-end video classification network should highlight
- Assessment of diverse DL architectures to identify the most promising one. The architectures varied on structure topology and video assessment strategy
- The data used to train the networks was collected from the emergency department (ED) of the Fondazione IRCCS Policlinico San Matteo Hospital in Pavia. The medical staff at the ED collected 12 clips for each patient and assigned each clip a standardised score using standard scales [16,17]. In total, data was collected from 450 patients, yielding a total of 5400 clips. However, not all clips were scored by the same medical practitioner, so the ED staff conducted a review to ensure that all clips had accurate and standardised scores, avoiding discrepancies that have been reported in other studies
- Three different ranking scales to assess the severity of lung involvement, whereas the literature proposes investigations whose classifications mainly retain whether or not there is a viral pneumonia
- Robustness to noise and adversarial attacks assessed through a data augmentation process applied to the training set
- Eventually, we assessed the X3D architecture concerning t-SNE, PCA, and Grad-Cam strategies to demonstrate the trustworthiness of the results

We organised the paper as follows: Materials and Methods describe the AI methodologies, algorithms, and data used to conduct the experiments in detail. Results and Discussion retain the essential results to compare our study with the literature, thus emphasising their significance. 

Eventually, the last section offers the main scientific advancements that extend the field based on current knowledge and our achievements.

**Figure 1:**
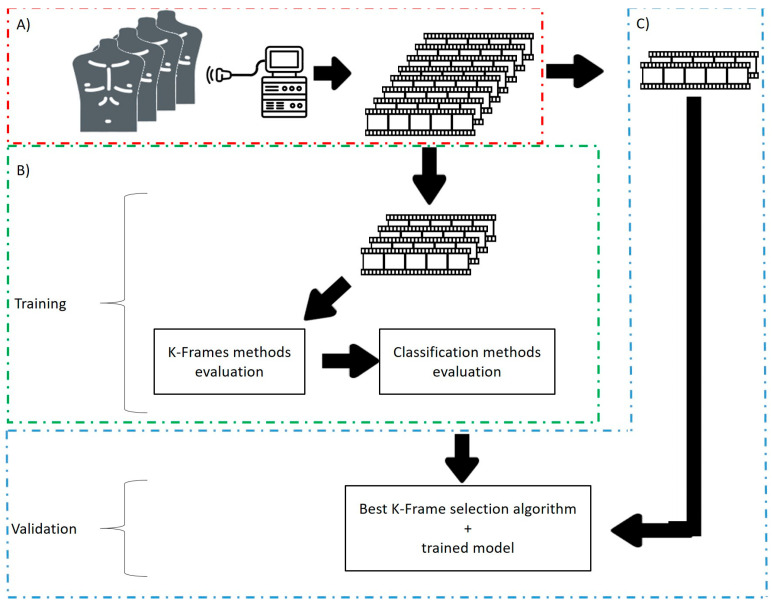
Main steps of the proposed work. (**A**) Data are acquired in the Emergency Department of the San Matteo Hospital to create a dataset of Lung UltraSound clips. (**B**) Part of these clips are selected as training set. These videos are also augmented to enlarge the training set. In this phase, different key-frames selection algorithm and classification techniques are evaluated. (**C**) The best key-frame selection algorithm and the best classifier are validated through the test set.

Figure 1 shows the main steps of the proposed work.

## 2. Materials and Methods

In this section, we provide a detailed description of the dataset we used for SARS-CoV-2 end-to-end video classification and the selection, design, and training of the CNN architecture we employed. Specifically, we focus on data augmentation, transfer learning, training options, and the hyperparameters used to train and fine-tune our video classification networks.

In particular, we addressed end-to-end video classification, since the proposed system takes as input the original clip acquired by the medical instrumentation. Then, the classification system elaborates this video and gives as output the pneumonia severity classification. The elaboration consists of two main steps: the former is the video summarisation, which produces a shorted clip containing only the most informative frames, while the latter is the classification of this summarised video adopting a suitable deep learning architecture.

### 2.1. Lung Ultrasound Score

The first step to analysing the LUS dataset is understanding the manuscript’s scoring methodology. Remarkably, this investigation employed the ranking scale we introduced in our prior study that laid the foundations for this improvement. Table 1 summarises how physicians assessed the severity of lung involvement by assigning patients’ lung portions with a standardised score. The description explains what deep video classification architectures concentrate on.

This manuscript operates at most four classes to indicate the severity of lung involvement. The pneumonia severity classification scale comprises scores ranging from 0 to 3, where Score 3 describes a lung almost incapable of breathing. A lung rated as Score 3 indicates that the illness affects the pleural line, namely the interface between the fluid-rich soft tissues of the wall and the gas-rich lung tissue [18], whilst Score 0 identifies a healthy lung portion.

### 2.2. SARS-CoV-2 LUS Dataset

Since March 2020, medical personnel at the San Matteo Hospital’s ED have been gathering LUS tests to examine the health of patients with suspected SARS-CoV-2 infection. The doctors operated the Aloka Arietta V70 ultrasound device (Hitachi Medical Systems), which works with convex and linear probes at frequencies of 5 MHz and 12 MHz, respectively. They standardised the procedure by focusing on the pleural line at a depth of 10 cm with the convex probe, and adjusting the gain to optimise the imaging of the pleural line, vertical artifacts, and peripheral consolidations with or without air bronchograms. Longitudinal and transversal scans were performed to examine the full length of the pleural line, with all harmonics and artifact-erasing software disabled.

LUS was performed on patients with suspected SARS-CoV-2 infection due to the potential presence of false negatives in rRT-PCR testing. Specifically, the artefacts observed in the earlier section of the manuscript may be caused by either pulmonary edema or non-cardiac causes of interstitial syndromes [19]. Even if a swab test is negative, patients with lung involvement have a high likelihood of being SARS-CoV-2 positive. Medical practitioners are trained to distinguish suspected cases from healthy patients using a triaging process that includes LUS examination.

In this study, a “clip” refers to the result of an LUS test, consisting of a set of frames or images. The medical personnel at the hospital collected 12 clips for each patient, all assigned a standardised LUS score according to Table 1 [16,17]. Data was collected from 450 patients treated in Pavia, yielding a total of 5400 clips. Table 2 lists the subjects classified as SARS-CoV-2 positive and negative, along with their clinical data in the form of median and 25th–75th percentile values. The LUS Score entry indicates the sum of the values obtained from the 12 examinations for each patient.

Nonetheless, different doctors scored the clips, so the ED staff conducted a review to validate the classifications and avoid incorrect severity-scoring issues. This process ensured that each clip had a standardised rank value and that there were no discrepancies in the scores associated with different clips at the same severity stage, as emphasised in other studies [20].

Accordingly, the dataset operated in this manuscript consisted of 624 clips randomly selected from the initial 5400 clips distribution.

**Figure 2:**
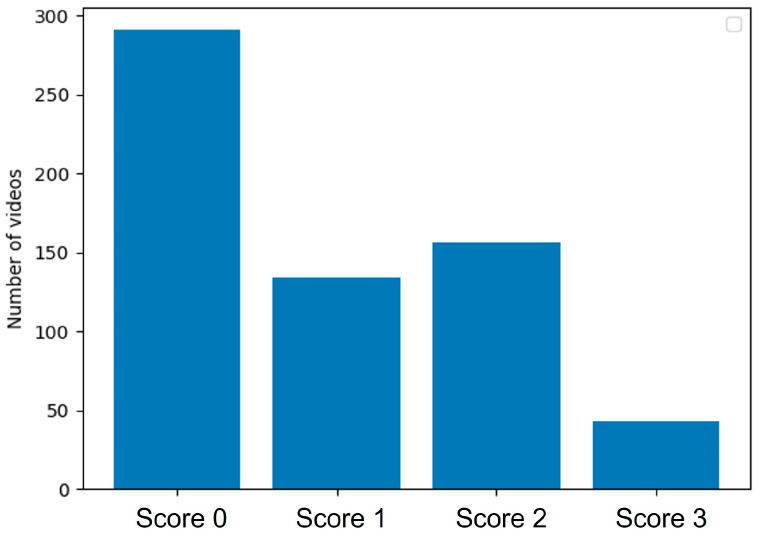
Dataset classes distribution.

Fondazione IRCCS Policlinico San Matteo Hospital’s Emergency Department physicians oversaw the methodical procedure and ensured that the labeling was accurate. During the first part of the data collection and annotation process, they manually selected all clips from each patient, assessed the quality of each clip, and either proceeded to evaluate it based on the two scoring methodologies or discarded it. They reviewed each clip to assign a score and verify that SARS-CoV-2 pneumonia patterns, as described in Section 2.1, were present. The patient selection process was random and blinded to reject the hypothesis of biased outcomes. Some subjects may have received fewer LUS exams than others due to the detection of severe lung involvement in the early stages of the procedure. The entire annotation and collection process took longer than one month, resulting in 624 gathered clips based on the initial 5400 clips. Figure 2 shows the class distribution in the dataset, divided into four tiers.

Finally, this study randomly split the data into training (80%), validation (10%), and testing (10%) sets, following standard deep learning practice [21] and keeping the training set size as small as possible to reject the overfitting hypothesis.

Similarly, we increased the statistical variance in the training set by applying various data augmentation strategies listed in Table 2, which forced the networks to focus on relevant information. We applied geometric, filtering, random center cropping, and color transformations to the training frames. The literature demonstrated that these methods are effective when applied to SARS-CoV-2 [22] and produce strong results in deep learning classification tasks, significantly reducing overfitting [23]. In addition, we added salt-and-pepper white noise to expand the training set. The X3D pre-trained architecture requires 3 × 32 × 224 × 224 clips in the PyTorch framework, so we converted the grayscale ultrasound frames to RGB to enable color augmentation. Data augmentation modifies the training data numerically, introduces statistically diverse samples, and enables the architectures to robustly classify new frames. The data augmentation process shifts the frame’s point of interest, slightly modifying its shape or color along with noise, preparing the models to expect relevant features to be in a different location. The models also learn to reject disruptions such as probe sensor measurement errors. Therefore, this research applied augmentations to all training images, regardless of the probe used for the LUS examination.

We repeatedly applied augmentations to the training data to exponentially expand the training set.

According to the AI act established by the European Commission, ensuring cybersecurity is crucial in guaranteeing that artificial intelligence applications are resistant to attempts to alter their service, behavior, and performance, or compromise their safety properties through malicious interference by third parties exploiting system vulnerabilities. Cyberattacks on AI systems can leverage assets specific to AI, such as introducing adversarial attacks on trained models, namely providing the optimised architectures with slightly different inputs and confuse their behavior. Consequently, providers of high-risk AI systems should take appropriate measures to ensure an appropriate level of cybersecurity in relation to the risks, taking into account the underlying ICT infrastructure as necessary.

At the end of the training settings management stage, with transfer learning and data augmentation, we collected 29,952, 171, and 171 clips for the training, validation, and test sets.

### 2.3. Key-Frame Selection Algorithm

There are several ways to summarise video data, including selecting the most important frames, reducing the memory needed for video processing and storage, and simplifying the structure of the video information. This paper used three different methods for extracting key frames:

1. Histogram [24]: a histogram approach that compares the difference of consecutive frames to a threshold value
2. Relative entropy [25]: a method based on relative entropy, a measure of the distance between probability distributions in information theory to calculate the distance between neighboring video frames and partition a video sequence [26]
3. ResNet + K-means clustering [27]: a technique that involves using a ResNet-18 to encode the information in each frame, followed by K-means clustering to sample frames from groups and produce an unsupervised video summary. The ResNet employed in this method is the standard ResNet18 architecture which is a 72 layers network with 18 deep layers.

All these key-frames selection algorithms have been used to summarise the clips. These summarised clips are then used to train the networks described in Section 2.4. It is important to highlight that, in the classification phase, the key-frame selection represents the step that produces the input for the trained network.

### 2.4. Video Classification Architecture

This research evaluated various architectures to perform the end-to-end video classification of the data described in the earlier section. This investigation aims to determine the severity of lung involvement in an end-to-end fashion operating three different hierarchical ranking scales. All the architectures receive the summarisation of clips evaluated through the three methodologies we described in Section 2.3. Remarkably, we evaluated the following architectures:

1. CNN + LSTM [28]: we designed the first architecture leaning on features extracted from a CNN, specifically a residual architecture, concerning each clip’s frame. Accordingly, we treated the sequence of features belonging to the frames as a time series processed through the LSTM network. The CNN network is the same adopted in [28] while we considered a single LSTM cell.
2. CNN + Transformer [29]: the second architecture follows the idea mentioned earlier. Accordingly, the CNN section remains unvaried, but we replaced the LSTM with an attention-based transformer for sequence classification coming from Natural Language Processing (NLP) applications.
3. R(2+1)D [30]: researchers usually employ this convolutional neural network for action recognition that employs R(2+1)D convolutions in a ResNet-inspired architecture. The use of these convolutions over regular 3D Convolutions reduces computational complexity, prevents overfitting, and introduces more non-linearities that allow for better functional relationships. The R(2+1)D adopted in this work features 5 (2+1)D convolutional layers followed by a fully connected network.
4. Multiscale Vision Transformer (MViT) [31]: can classify videos joining multiscale feature hierarchies with transformer models. MViTs have several channel-resolution scale stages. Starting from the input resolution and a small channel dimension, the stages hierarchically expand the channel capacity while reducing the spatial resolution. This growth process creates a multiscale pyramid of features with early layers operating at a high spatial resolution to model simple low-level visual information and deeper layers at spatially coarse but complex, high-dimensional features. In this case we adopted the MViT_base_32 × 3 which concurrently elaborates 32 frames.
5. Slow-fast architecture [32]: the architecture presents a novel method to analyse the contents of a video segment. The architecture’s core comprises two parallel convolution neural networks (CNNs) on the same video segment—a Slow and a Fast pathway. The authors observed that frames in video scenes usually contain two distinct parts—static areas in the frame, which do not change at all or change slowly, and dynamic areas, which indicate something important that is currently going on.
6. X3D [33]: it is a family of efficient video networks that progressively boost a tiny 2D image classification architecture along multiple network axes in space, time, width and depth. Motivated by feature sampling methods in machine learning, a stepwise network growth approach extends a single axis in each step, such that sound accuracy to complexity trade-off exists. The X3D advantages include that despite having a high spatiotemporal resolution, it is incredibly light in terms of network width and parameters. In particular, we adopted the model X3DM from [33].

### 2.5. Performance Evaluation

First, we assessed the quality of the key-frame video summary algorithms. This research represents a follow-up of the first study concerning frame classification [8]. Hence, we have a carefully set of selected frames containing the exact patterns in Table 1. Nonetheless, video summaries also produce transition frames due to probe movements or noise. Hence, only some of the frames contain patterns. This manuscript aims to deliver end-to-end video classification without manual frame extraction from expert professionals. Hence, we trained residual architectures on the dataset retained from extracting key-frames via all the algorithms described in Section 2.3. We tested the networks on frames carefully extracted from the Fondazione IRCCS Policlinico San Matteo ED’s medical personnel. We cannot retain 100% accuracy on the test set due to the presence of transition frames. However, good classification performance on the test set implies that it is similar to the training one, thus retaining the patterns in Table 1.

Similarly, we evaluated the following similarity indexes (Equations (1)–(3)) to compare the datasets originating from the key-frame algorithms (Section 2.3) and the frames derived from the first study, which laid the grounds for this, carefully selected from San Matteo ED’s skilled physicians.

(1) $$SSIM=\frac{\begin{array}{cc} \left(2\;\mu_{x}\mu_{y}+c_{1}\right) & \left(2\sigma_{xy}+c_{2}\right) \end{array}}{\begin{array}{cc} \left(\mu_{x}^{2}+\mu_{y}^{2}+c_{1}\right) & \left(\sigma_{x}^{2}+\sigma_{y}^{2}+c_{2}\right) \end{array}}$$

(2) $$D_{KL}(P||Q)=\sum_{𝓍\in \chi }P\left(x\right)log\left(\frac{P\left(x\right)}{Q\left(x\right)}\right)$$

(3) $$JSD\;(P||Q)=\frac{1}{2}D_{KL}\left(P||\frac{P+Q}{2}\right)+\frac{1}{2}D_{KL}\left(Q||\frac{P+Q}{2}\right)$$

Equation (1) reports the Structural Similarity Index (SSIM) where $\mu_{x}$ and $\mu_{xy}$ are the pixel sample mean of image x and y, respectively. The terms $\sigma_{x}^{2}$ and $\sigma_{y}^{2}$ are the variance of image x and y, respectively. Finally, $\sigma_{xy}$ is the covariance of the two images and $c_{1}$ and $c_{2}$ are factors used to stabilise the division with a weak denominator [34].

Equation (2) is the Kullback-Leibler Divergence ($D_{KL}$) where P and Q are two discrete probability distributions over the same probability space χ.

Finally, Equation (3) defines the Jensen-Shannon divergence as a symmetrised and smoothed version of Equation (2).

It is crucial to decrease false negatives to the maximum extent feasible, particularly when treating an infectious disease such as SARS-CoV-2. A patient’s incorrect diagnosis introduces a false negative, which causes improper care, lack of necessary treatment that reflects cross-contamination among subjects with additional pathologies, and faulty medications that may harm an infected person.

In this research, the performance of the network classifications was evaluated using the validation and test sets. The focus was not only on accuracy, but also on precision, recall, and F1-Score (Equations (4)–(7)) and ROC-AUC [35]. These metrics, defined in the equations below, were calculated for each category for all classification tasks. The importance of reducing false negatives, particularly in the context of infectious diseases such as SARS-CoV-2, cannot be overemphasised. False negatives can lead to incorrect diagnoses, inadequate treatment, cross-contamination among patients with other pathologies, and potentially harmful medications. True Positive (*TP*) refers to correct classifications, False Negative (*FN*) refers to incorrect classifications, True Negative (*TN*) refers to correct classifications, and False Positive (*FP*) refers to incorrect classifications.

(4) $$Accuracy=\frac{TN+TP}{TN+FP+TP+FN}$$

(5) $$Precision=\frac{TP}{TP+FP}$$

(6) $$Recall=\frac{TP}{TP+FN}$$

(7) $$F1-Score=\frac{2*Precision*Recall}{Precision+Recall}\;$$

Researchers often place a particular emphasis on recall in order to reduce false negatives. Recall measures the performance of correctly identifying frames that do not contain SARS-CoV-2 pneumonia patterns and that belong to either of the considered classes or that display a healthy lung. Precision tells the reader about the classification performance in detecting the considered patterns. Therefore, the F1-Score is considered as a function of the previous two metrics. This parameter provides a more accurate measurement in terms of accuracy, taking into account the trade-off between precision and recall in unbalanced class distributions. Therefore, we need to evaluate recall and F1-Score in order to minimise false negatives while maintaining high precision.

Eventually, this study assessed the quality and robustness of the classification performance through explainable AI strategies. Accordingly, we operated the gradient class activation mapping (Grad-CAM) algorithm and the statistical analysis of features deriving from deep architectural layers to evaluate whether we could clearly identify patterns from LUS clips and how dividable such patterns are in architectural encoded features. The former enables the interpretation of the architecture’s decision-making. Indeed, it emphasises the decisive parts to assign a rank through a heat map. Concerning the latter strategies, we performed PCA and t-SNE over the inner layers of the X3D architecture. Hence, we coloured the clusters according to the clips’ original classes.

## 3. Results

The study assessed the quality of video summaries produced by the key-frame selection algorithms. Table 3 contains the performance of residual architectures, trained on the dataset of extracted key-frames, tested on the set of frames derived from the first study, which laid the foundations for this manuscript, carefully selected by San Matteo ED’s expert physicians.

Likewise, we report in Table 4 the similarity indexes to compare the summarised frames and the ones selected by the San Matteo ED’s medical personnel. Table 4 shows the similarity between the automatically selected frames and the ones manually chosen by an expert medical practitioner. We expected the measurements to slightly diverge from the ideal values because extracted frames contain transition information such as probe movements or patient’s respiratory motion. Table 4 highlights two main results. The first is that the three key-frame selection methods feature similarity indexes values that are very close, meaning that the extracted frames are nearly the same. The latter is that the distance from the original dataset is nearly the same for all the methods; therefore the summarisation performed by these algorithms is comparable in terms of informative content.

Hence, this research reports key-frame selection as a promising methodology to deliver the LUS clips summarisation and reduce memory footprint, enabling faster training.

Being that the three similarity indexes are very close, we also adopt the three key-frames selection algorithms to enlarge the size of our dataset.

We employed a ResNet-50 to measure the closeness between the frames extracted by the doctors and the automatic algorithms. Namely, we trained the architecture only with the automatically extracted frames to classify the manually chosen frames by the doctor (at the end of the random selection process described in Section 2.2). Therefore, we exploited overfitting as a measure to understand how well the automatic methods reproduce the statistical distribution of frames containing SARS-CoV-2 pneumonia patterns. Table 5 contains the classification results exceeding 90%, thus highlighting the trustworthiness of our results. In fact, the high values featured by all the metrics can be obtained only if the training set features are similar to the validation set ones. In other words, these values clearly indicate that the frame automatically extracted by the proposed method are close to the ones manually selected by the doctors.

The investigation assessed the architectures’ diagnostic performance starting from the binary classification, which consists of evaluating all the scores in Table 1 as SARS-CoV-2 positive. Hence, the classes are simply either healthy or infected. Retaining bad diagnostic outcomes in this classification task prevents the models from behaving correctly in multi-class scenarios. We also discard the wrong video summary extraction hypothesis from the algorithms in Section 2.3 since the doctors assessed the presence of the patterns in Table 1.

We report that the first five architectures mentioned in Section 2.4 yielded binary accuracy ranging from 30% to 60% at most. Accordingly, we ended the experiments on such architectures. On the other hand, the X3D architecture retained good performance in Table 3, and we continued the research operating only this latter deep neural network. Table 6 shows a comparison between X3D, R(2+1)D and MViT considering the different key-frames algorithms described in Section 2.3 and the binary, three-way, and four-way classifications. The results are reported in terms of mean values.

Table 1 reports the severity scale this manuscript employed to assess the severity of lung involvement. Accordingly, we evaluated the following classification tasks:

1. Binary classification: only two classes exist, namely Score 0 and the set resulting from the union of all the other scores
2. Three-way classification: we consider Score 1 and Score 2 as a unique rank, thus scoring lungs as either healthy (Score 0), containing B-lines (Score 1 or Score 2) or consolidations (Score 3)
3. Four-way classification: we considered all the scores in Table 1

Hence, this manuscript reports the X3D architecture’s results according to the three classification tasks mentioned above. The designated architecture steadily approached optimisation convergence concerning the hyperparameters and training options in Table 7.

The results of the network’s weights at the end of each training process are reported, regardless of the number of epochs chosen for optimisation. We did not perform early stopping, which involves evaluating the epoch that shows promising performances with the validation set during optimisation, because the training process converged when the number of epochs elapsed. In addition, the training settings in Table 7, which contain extensively tuned hyperparameters to achieve recall and F1-Score above 90%, are reported. This indicates a reliable balance between precision and recall, which is important when working with unbalanced datasets (Figure 2).

Table 3 shows the X3D video classification network, which performs exceptionally well in all scenarios when provided with the LUS clip summaries using the key-frame selection algorithms, and achieves excellent results in the four-way classification task. It also shows an average recall of over 89%, demonstrating the effectiveness of the classification for detecting SARS-CoV-2 pneumonia patterns.

**Figure 3:**
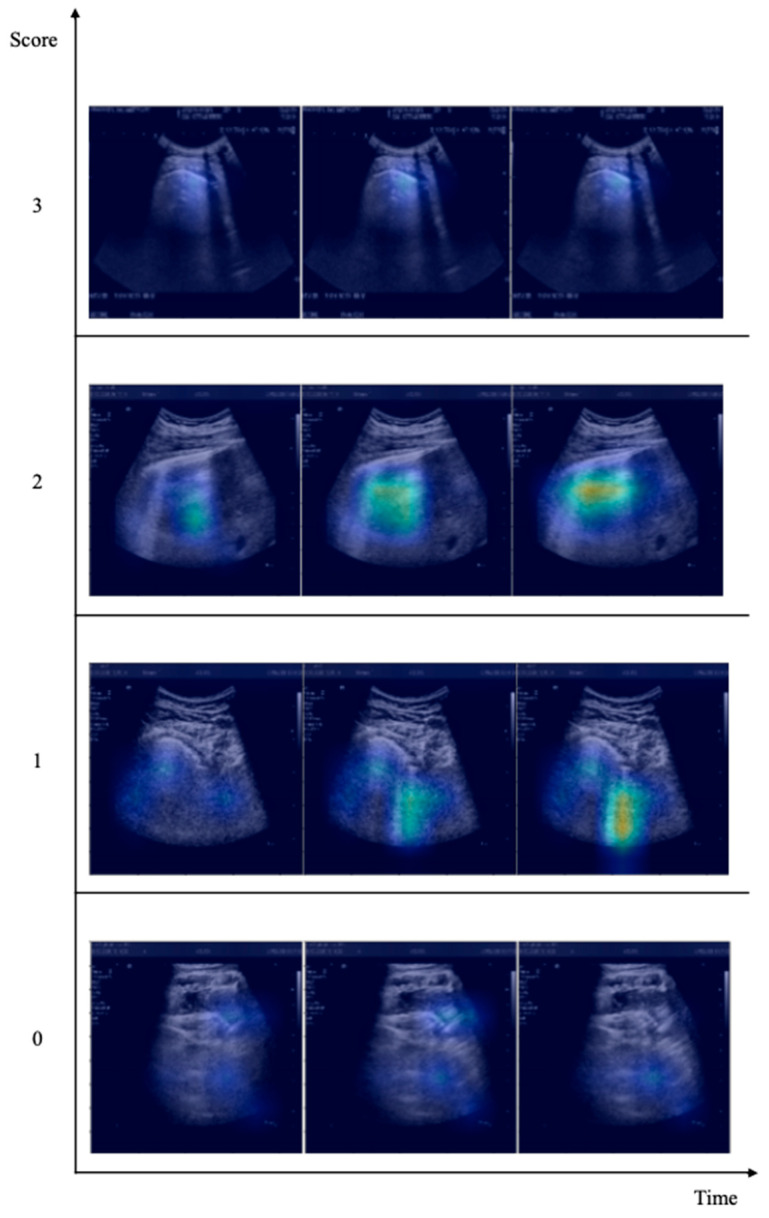
X3D network Grad-CAM results. The numbers on the vertical axis indicate the score associate to each video, while on the time axis three significant video frames are shown. The Grad-CAM activation map is superimposed to each frame, highlighting the image regions which are important for the classification.

We used Grad-CAM to validate the network’s decision-making. The physicians evaluated whether the X3D correctly identified B-lines, pleural line abnormalities, or other patterns examined in the LUS scoring section, which is the procedure doctors use to assess patients’ health. Figure 3 shows the behavior of X3D in the most complex four-way classification task. We present the Grad-CAM results starting from the lowest score, indicating that the subject being considered is healthy, and approaching the highest score, indicating that the patient requires urgent respiratory assistance. The architecture accurately and thoroughly highlights all patterns, including A and B lines and small or large consolidations.

**Figure 4:**
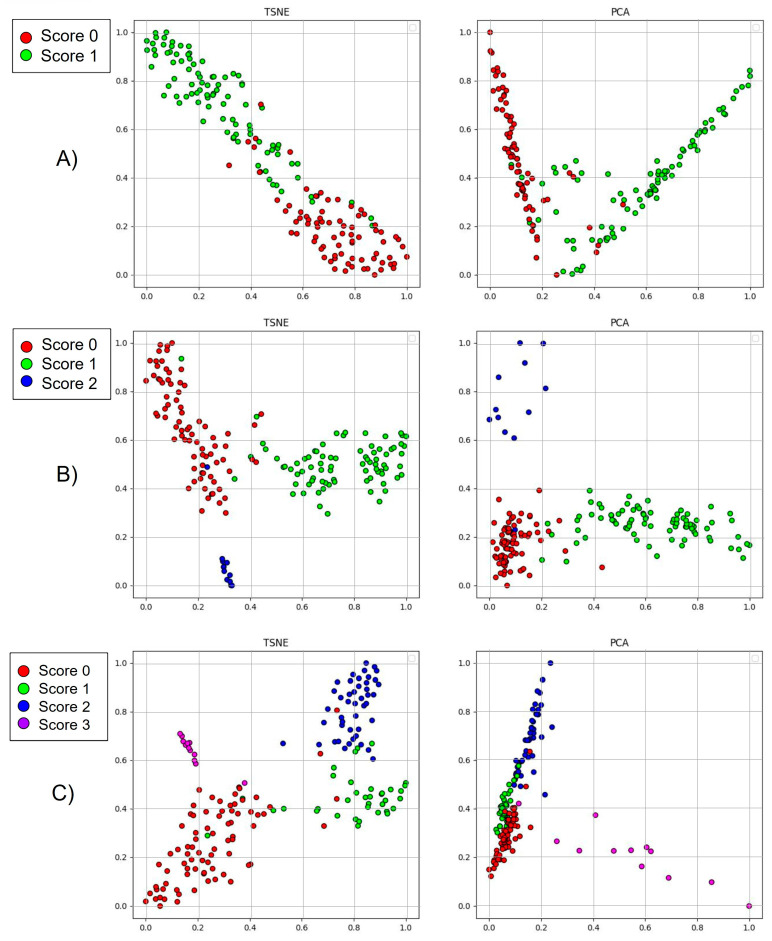
t-SNE and PCA results: (**A**) binary classification, (**B**) three-way classification, and (**C**) four-way classification.

Eventually, we further validated the results by analysing the features extracted from the X3D architecture related to each clip coming from the test set. We reduced the order of each feature operating t-SNE and PCA algorithms in 2D. Figure 4 displays the results coloured concerning the original class of each clip. Hence, distinct clusters originate from the features extracted from the network concerning each clip, emphasising that the X3D can discriminate between the SARS-CoV-2 patterns to rank the severity of lung involvement and robustness to adversarial attacks. In particular, Figure 4A shows the t-SNE and PCA results related to the binary classification. The red and green points constitute two different clusters in the bidimensional plane, meaning that the features extracted by the network can discriminate between the two classes. Similar considerations can be made for Figure 4B,C. In these cases, the only difference is in the number of classes considered for the classifications, which reflects an equivalent number of clusters in the charts.

## 4. Discussion

Table 8 reports the literature results. First, we must stress that we do not consider an end-to-end classification task extracting features from a CNN architecture later ensembled into a sequence to be processed by an architecture such as an LSTM. Accordingly, the last two investigations in Table 8 do not assess LUS clips in an end-to-end manner but either perform the action mentioned earlier or the frame classification. On the other hand, the first research assesses LUS clips employing the I3D architecture. 

Furthermore, the first investigation is the only one assessing the clips operating one of the tasks we mentioned in this manuscript, namely the three-way classification. All the others discriminate between bacterial or viral (SARS-CoV-2) pneumonia and healthy lungs.

Hence, our results in Table 3 improve the results proposed by the literature by operating diverse classification tasks that assess the severity of lung involvement and by employing the key-frame selection algorithm joined with an end-to-end X3D video classification architecture. 

In conclusion, previous research on SARS-CoV-2 LUS clip assessment has some limitations. Some studies only utilised transfer learning and relied on low quality data sources that were not assessed by a qualified physician. Additionally, only the first study in Table 8 used a severity scale to diagnose LUS clips and assess patients’ health. It employed an explainable algorithm to validate the network’s decision-making. The other studies attempted to distinguish between different types of pneumonia and applied image classification networks with minor modifications to analyze small clip sections. In this study, we propose a straightforward approach to apply DL to LUS clips and assess the severity of SARS-CoV-2 pneumonia. We utilised a pre-trained video classification architecture in three classification tasks and utilised an existing and validated ranking scale. It helps differentiate between cardiogenic and non-cardiogenic causes of B-lines [19] and enables the early detection and timely treatment of ARDS pneumonia symptoms. At the time of writing, this is the first study to evaluate the end-to-end LUS clip assessment using the scoring methodologies listed in Table 1. Specifically, we validated our collection of clips using data augmentation, transfer learning, and hyperparameter tuning to obtain the results presented in this paper.

## 5. Conclusions

In summary, we developed a reliable AI diagnostic tool to provide overworked medical personnel with an efficient and affordable SARS-CoV-2 detection system. The close collaboration with the Fondazione IRCCS Policlinico San Matteo ED allowed us to conduct our research using highly reliable and validated LUS data. We employed modern DL strategies, including video classification architectures, data augmentation, transfer learning, and key-frame selection algorithms, to assess the severity of lung involvement in SARS-CoV-2 infected individuals.

This study used three different scoring scales to measure accurate and robust diagnostic performance. We addressed the issue of data heterogeneity, including low sensitivity leading to inadequate treatment and cross-contamination. We improved existing state-of-the-art diagnostic methods [20,35,36] for detecting SARS-CoV-2 in LUS clips.

This study provides an end-to-end approach for classifying and scoring LUS clips. The Fondazione IRCCS Policlinico San Matteo ED reviewed every exam to uniformly assign the same score to lungs with the same disease stage.

The proposed AI diagnostic tool features the advantage of providing a tool to detect and diagnose SARS-CoV-2 severity only considering LUS. Therefore, this analysis produced a DL-based system to automatically detect SARS-CoV-2 pneumonia patterns in LUS clips and rate their severity based on three standardised scoring scales with impressive, reliable, and promising results. The main drawback is that ultrasound imaging technologies require specialised expertise to achieve diagnostic reliability, including high sensitivity and overall accuracy. Moreover, the validated results are lacking an external cohort of patients, but this problem could be easily solved in the future by involving other hospitals into the research.

## Figures and Tables

**Table 1:** The pneumonia severity scale [8].

Severity Score	Description
Score 0	A-lines with at most two B-lines
Score 1	Artefacts occupy at most 50% of the pleura
Score 2	Artefacts occupy more than 50% of pleura, consolidated areas might be visible
Score 3	Tissue-like pattern

**Table 2:** Data augmentation methods adopted in this work.

Augmentation Method	Description
Frames resize	Resize every frame of the video to size of 224 × 224
Random rotation	Rotate randomly every frame between −10 to 10 degrees
Random translate	Translate randomly every frame, either vertical or horizontal
Image noise	Adds salt-and-pepper noise to frames. Namely, random pixels get randomly coloured towards white

**Table 3:** Residual architectures performance on the validation set.

Metric	Bynary Classification		Three-Way Classification			Four-Way Classification			
	Class 0	Class 1	Class 0	Class 1	Class 2	Class 0	Class 1	Class 2	Class 3
Accuracy	0.859	0.942	0.972	0.919	0.923	0.925	0.838	0.930	0.909
Precision	0.859	0.942	0.972	0.919	0.923	0.925	0.838	0.930	0.909
Recall	0.936	0.871	0.897	0.975	1.000	0.949	0.861	0.889	0.833
F1-Score	0.896	0.905	0.933	0.946	0.960	0.937	0.849	0.909	0.870
ROC-AUC	0.967	0.967	0.986	0.987	1.000	0.982	0.978	0.903	0.998

**Table 4:** Similarity indexes.

Dataset	Original											
	Class 0			Class 1			Class 2			Class 3		
	SSIM	D_KL_	JSD	SSIM	D_KL_	JSD	SSIM	D_KL_	JSD	SSIM	D_KL_	JSD
Original	0.41	0.18	0.23	0.57	0.43	0.21	0.59	0.16	0.20	0.41	0.06	0.14
Histogram	0.27	2.34	0.70	0.36	2.39	0.69	0.30	18.00	0.81	0.31	5.83	0.80
Relative entropy	0.26	3.70	0.79	0.36	6.08	0.82	0.36	15.59	0.81	0.32	1.70	0.66
Resnet + K-means	0.26	2.71	0.60	0.37	0.63	0.40	0.31	6.91	0.80	0.31	9.61	0.83

**Table 5:** ResNet-50 classification performance on the dataset of validated SARS-CoV-2 pneumonia frames.

Metric	Bynary Classification		Three-Way Classification			Four-Way Classification			
	Class 0	Class 1	Class 0	Class 1	Class 2	Class 0	Class 1	Class 2	Class 3
Accuracy	0.859	0.942	0.963	0.981	0.989	0.921	0.999	0.968	0.994
Precision	0.859	0.942	0.963	0.981	0.989	0.921	0.999	0.968	0.994
Recall	0.936	0.871	0.952	0.976	1.000	0.976	1.000	0.939	0.984
F1-Score	0.896	0.905	0.958	0.979	0.995	0.948	1.000	0.954	0.989
ROC-AUC	0.967	0.967	0.998	0.999	1.000	0.996	1.000	0.955	0.999

**Table 6:** Comparison between X3D, R(2+1)D, and MViT. The metrics have been computed as mean values across the classes considered for the classification. The best results are highlighted in bold.

Key-Frame Selection	Metric	X3D			R(2+1)D			MViT		
		Binary Classification	Three-Way Classification	Four-Way Classification	Binary Classification	Three-Way Classification	Four-Way Classification	Binary Classification	Three-Way Classification	Four-Way Classification
Entropy	Accuracy	**0.773**	**0.739**	**0.687**	0.272	0.196	0.448	0.272	0.158	0.053
	Precision	**0.773**	**0.739**	**0.687**	0.272	0.196	0.448	0.272	0.158	0.053
	Recall	**0.775**	**0.778**	**0.632**	0.500	0.367	0.494	0.500	0.333	0.250
	AUC	**0.814**	**0.865**	**0.877**	0.500	0.640	0.731	0.500	0.499	0.502
	F1-Score	**0.772**	**0.756**	**0.655**	0.352	0.255	0.425	0.352	0.214	0.087
Histogram	Accuracy	**0.770**	**0.845**	**0.654**	0.272	0.272	0.225	0.272	0.158	0.018
	Precision	**0.770**	**0.845**	**0.654**	0.272	0.272	0.225	0.272	0.158	0.018
	Recall	**0.772**	**0.625**	**0.609**	0.500	0.404	0.387	0.500	0.333	0.250
	AUC	**0.867**	**0.837**	**0.815**	0.500	0.693	0.590	0.442	0.429	0.490
	F1-Score	**0.771**	**0.659**	**0.615**	0.352	0.309	0.267	0.352	0.214	0.033
ResNet + K-means	Accuracy	**0.862**	**0.843**	**0.743**	0.272	0.158	0.452	0.272	0.158	0.066
	Precision	**0.862**	**0.843**	**0.743**	0.272	0.158	0.452	0.272	0.158	0.066
	Recall	**0.855**	**0.840**	**0.688**	0.500	0.333	0.402	0.500	0.333	0.250
	AUC	**0.934**	**0.934**	**0.826**	0.500	0.564	0.674	0.358	0.321	0.504
	F1-Score	**0.858**	**0.840**	**0.695**	0.352	0.214	0.383	0.352	0.214	0.104

**Table 7:** Hyperparameters and training options for the X3D network.

Options and Hyper-Parameters	Two Classes	Three Classes	Four Classes
Initial Learning Rate	0.001	0.001	0.001
Learning Rate’s Drop Factor	0.5	0.5	0.5
Learning Rate’s Drop Period (Epochs)	3	3	3
Batch Size	2	2	2
L2—Regularisation	0.0001	0.0001	0.0001
Epochs	12	12	12
Environment	Single-GPU	Single-GPU	Single-GPU
Optimiser	Adam	Adam	Adam
Loss Function	Cross-Entropy	Cross-Entropy	Cross-Entropy

**Table 8:** Related works performance.

	Work		
	[13]	[14]	[15]
F1-Score	87–94%	58–95%	97%
Accuracy	N. A.	N. A.	96–89%
Precision	87–96%	66–100%	N. A.
Recall	86–92%	52–97%	98%
Performed task	Three-way classification	Discrimination between bacterial pneumonia, healthy and SARS-CoV-2	Discrimination between bacterial pneumonia, healthy and SARS-CoV-2
End-to-end video classification	Yes	No	No

## Data Availability

The datasets generated during the current study are available, under reasonable request.

## Abbreviations

Acronym	Meaning
ARDS	Acute Respiratory Distress Syndrome
CNN	Convolutional Neural Network
CT	Computed Tomography
CXR	Chest X-rays
DL	Deep Learning
ED	Emergency Department
FN	False Negative
FP	False Positive
Grad-CAM	Gradient Class Activation Mapping
LSTM	Long-Short Term Memory
LUS	Lung UltraSound
MLP	Multi-Layer Perceptron
MViT	Multiscale Vision Transformer
PCA	Principal Component Analysis
rRT-PCR	real-time Reverse Transcript-Polimerase Chain Reactions
TN	True Negative
TP	True Positive
ViT	Vision Transformer

## References

[1] Li Q., Guan X., Wu P., Wang X., Zhou L., Tong Y., Ren R., Leung K.S.M., Lau E.H.Y., Wong J.Y. (2020). Early Transmission Dynamics in Wuhan, China, of Novel Coronavirus–Infected Pneumonia. N. Engl. J. Med..

[2] Mohanty S.K., Satapathy A., Naidu M.M., Mukhopadhyay S., Sharma S., Barton L.M., Stroberg E., Duval E.J., Pradhan D., Tzankov A. (2020). Severe Acute Respiratory Syndrome Coronavirus-2 (SARS-CoV-2) and Coronavirus Disease 19 (COVID-19)—Anatomic Pathology Perspective on Current Knowledge. Diagn. Pathol..

[3] Shi H., Han X., Jiang N., Cao Y., Alwalid O., Gu J., Fan Y., Zheng C. (2020). Radiological Findings from 81 Patients with COVID-19 Pneumonia in Wuhan, China: A Descriptive Study. Lancet. Infect Dis..

[4] Soldati G., Smargiassi A., Inchingolo R., Buonsenso D., Perrone T., Briganti D.F., Perlini S., Torri E., Mariani A., Mossolani E.E. (2020). Proposal for International Standardization of the Use of Lung Ultrasound for Patients With COVID-19: A Simple, Quantitative, Reproducible Method. J. Ultrasound Med..

[5] Li Z., Yi Y., Luo X., Xiong N., Liu Y., Li S., Sun R., Wang Y., Hu B., Chen W. (2020). Development and Clinical Application of a Rapid IgM-IgG Combined Antibody Test for SARS-CoV-2 Infection Diagnosis. J. Med. Virol..

[6] Niederman M.S., Mandell L.A., Anzueto A., Bass J.B., Broughton W.A., Campbell G.D., Dean N., File T., Fine M.J., Gross P.A. (2001). Guidelines for the Management of Adults with Community-Acquired Pneumonia. Diagnosis, Assessment of Severity, Antimicrobial Therapy, and Prevention. Am. J. Respir. Crit. Care Med..

[7] Garg M., Prabhakar N., Bhalla A., Irodi A., Sehgal I., Debi U., Suri V., Agarwal R., Yaddanapudi L., Puri G. (2021). Computed Tomography Chest in COVID-19: When & Why?. Indian J. Med. Res..

[8] la Salvia M., Secco G., Torti E., Florimbi G., Guido L., Lago P., Salinaro F., Perlini S., Leporati F. (2021). Deep Learning and Lung Ultrasound for Covid-19 Pneumonia Detection and Severity Classification. Comput. Biol. Med..

[9] Chavez M.A., Shams N., Ellington L.E., Naithani N., Gilman R.H., Steinhoff M.C., Santosham M., Black R.E., Price C., Gross M. (2014). Lung Ultrasound for the Diagnosis of Pneumonia in Adults: A Systematic Review and Meta-Analysis. Respir. Res..

[10] Pagano A., Numis F.G., Visone G., Pirozzi C., Masarone M., Olibet M., Nasti R., Schiraldi F., Paladino F. (2015). Lung Ultrasound for Diagnosis of Pneumonia in Emergency Department. Intern. Emerg. Med..

[11] Bourcier J.E., Paquet J., Seinger M., Gallard E., Redonnet J.P., Cheddadi F., Garnier D., Bourgeois J.M., Geeraerts T. (2014). Performance Comparison of Lung Ultrasound and Chest X-ray for the Diagnosis of Pneumonia in the ED. Am. J. Emerg. Med..

[12] Manoj M.K., Atalla S., Almuraqab N., Moonesar I.A. (2022). Detection of COVID-19 Using Deep Learning Techniques and Cost Effectiveness Evaluation: A Survey. Front. Artif. Intell..

[13] Erfanian Ebadi S., Krishnaswamy D., Bolouri S.E.S., Zonoobi D., Greiner R., Meuser-Herr N., Jaremko J.L., Kapur J., Noga M., Punithakumar K. (2021). Automated Detection of Pneumonia in Lung Ultrasound Using Deep Video Classification for COVID-19. Inform. Med. Unlocked.

[14] Barros B., Lacerda P., Albuquerque C., Conci A. (2021). Pulmonary COVID-19: Learning Spatiotemporal Features Combining CNN and LSTM Networks for Lung Ultrasound Video Classification. Sensors.

[15] Rahhal M.M.A., Bazi Y., Jomaa R.M., Zuair M., Melgani F. (2022). Contrasting EfficientNet, ViT, and GMLP for COVID-19 Detection in Ultrasound Imagery. J. Pers. Med..

[16] Mongodi S., Bouhemad B., Orlando A., Stella A., Tavazzi G., Via G., Iotti G.A., Braschi A., Mojoli F. (2017). Modified Lung Ultrasound Score for Assessing and Monitoring Pulmonary Aeration. Ultraschall Med..

[17] Secco G., Delorenzo M., Zattera C., Moore B.G., Demitry L., Vezzoni G., Resta F., Barcella B., Cappa G., Perrone T. (2020). Lung Ultrasound in COVID-19: A Useful Diagnostic Tool. Emerg. Care J..

[18] Lichtenstein D.A. (2016). The Pleural Line. Lung Ultrasound Crit. Ill.

[19] Arntfield R., Vanberlo B., Alaifan T., Phelps N., White M., Chaudhary R., Ho J., Wu D. (2021). Development of a Convolutional Neural Network to Differentiate among the Etiology of Similar Appearing Pathological B Lines on Lung Ultrasound: A Deep Learning Study. BMJ Open.

[20] Roy S., Menapace W., Oei S., Luijten B., Fini E., Saltori C., Huijben I., Chennakeshava N., Mento F., Sentelli A. (2020). Deep Learning for Classification and Localization of COVID-19 Markers in Point-of-Care Lung Ultrasound. IEEE Trans. Med. Imaging.

[21] Islam M.M., Karray F., Alhajj R., Zeng J. (2021). A Review on Deep Learning Techniques for the Diagnosis of Novel Coronavirus (COVID-19). IEEE Access.

[22] Monshi M.M.A., Poon J., Chung V., Monshi F.M. (2021). CovidXrayNet: Optimizing Data Augmentation and CNN Hyperparameters for Improved COVID-19 Detection from CXR. Comput. Biol. Med..

[23] Shorten C., Khoshgoftaar T.M. (2019). A Survey on Image Data Augmentation for Deep Learning. J. Big. Data..

[24] Sheena C.V., Narayanan N.K. (2015). Key-Frame Extraction by Analysis of Histograms of Video Frames Using Statistical Methods. Procedia Comput. Sci..

[25] Guo Y., Xu Q., Sun S., Luo X., Sbert M. (2016). Selecting Video Key Frames Based on Relative Entropy and the Extreme Studentized Deviate Test. Entropy.

[26] Cover T.M., Thomas J.A. (2006). Elements of Information Theory.

[27] Yang S., Lin X. (2005). Key Frame Extraction Using Unsupervised Clustering Based on a Statistical Model. Tsinghua Sci. Technol..

[28] Abdullah M., Ahmad M., Han D. Facial Expression Recognition in Videos: An CNN-LSTM Based Model for Video Classification. Proceedings of the 2020 International Conference on Electronics, Information, and Communication, ICEIC.

[29] Xie Y., Zhang J., Shen C., Xia Y. (2021). CoTr: Efficiently Bridging CNN and Transformer for 3D Medical Image Segmentation. Lecture Notes in Computer Science, Proceedings of the International Conference on Medical Image Computing and Computer-Assisted Intervention, Strasbourg, France, 27 September–1 October 2021.

[30] Han X., Lu F., Yin J., Tian G., Liu J. (2022). Sign Language Recognition Based on R(2+1)D with Spatial-Temporal-Channel Attention. IEEE Trans. Hum. Mach. Syst..

[31] Fan H., Xiong B., Mangalam K., Li Y., Yan Z., Malik J., Feichtenhofer C. Multiscale Vision Transformers. Proceedings of the IEEE International Conference on Computer Vision.

[32] Wei D., Tian Y., Wei L., Zhong H., Chen S., Pu S., Lu H. (2022). Efficient Dual Attention SlowFast Networks for Video Action Recognition. Comput. Vis. Image Underst..

[33] Feichtenhofer C. X3D: Expanding Architectures for Efficient Video Recognition. Proceedings of the IEEE Computer Society Conference on Computer Vision and Pattern Recognition.

[34] Hastie T., Tibshirani R., Friedman J. (2009). The Elements of Statistical Learning.

[35] Baloescu C., Toporek G., Kim S., McNamara K., Liu R., Shaw M.M., McNamara R.L., Raju B.I., Moore C.L. (2020). Automated Lung Ultrasound B-Line Assessment Using a Deep Learning Algorithm. IEEE Trans. Ultrason. Ferroelectr. Freq. Control.

[36] Horry M.J., Chakraborty S., Paul M., Ulhaq A., Pradhan B., Saha M., Shukla N. (2020). COVID-19 Detection Through Transfer Learning Using Multimodal Imaging Data. IEEE Access.

